# Ethylenediaminetriacetic acid-functionalized activated carbon derived from *Posidonia oceanica* for the removal of Cu(ii)

**DOI:** 10.1039/d6ra06504j

**Published:** 2026-10-05

**Authors:** Hanedi Elhleli, Najib Mnasri, Salah Neghmouche Nacer, Mohammed Larbi Benamor, Ramzi Khiari, Younes Moussaoui

**Affiliations:** a University of Gafsa, Faculty of Sciences of Gafsa, Laboratory for the Application of Materials to the Environment, Water and Energy Tunisia; b University of Gafsa, Faculty of Sciences of Gafsa Tunisia younes.moussaoui@fsgf.ugaf.tn; c University of El Oued, Faculty of Exact Sciences, Chemistry Department El Oued Algeria; d University of El Oued, Faculty of Exact Sciences, Department of Chemistry, VTRS Laboratory El Oued Algeria; e Department of Textile, Higher Institute of Technological Studies of Ksar Hellal Ksar Hellal 5070 Tunisia; f University of Grenoble Alpes, CNRS, Grenoble INP, LGP2 Grenoble F-38000 France; g University of Sfax, Faculty of Sciences of Sfax, Organic Chemistry Laboratory (LR17ES08) Tunisia

## Abstract

*Posidonia oceanica*, a Mediterranean marine plant, was used in this research to prepare activated carbon. The carbon generated underwent functionalization with ethylenediaminetriacetic acid using *N*-[(3-trimethoxysilyl)propyl]-ethylenediaminetriacetic acid. The materials were characterized and then used as absorbents to remove copper(ii) ions from water. The Brunauer–Emmett–Teller surface areas were found to be 363 and 346 m^2^ g^−1^ for activated carbon and functionalized activated carbon, respectively. Adsorption studies were performed under several experimental conditions, such as varying adsorbent dosage, pH, temperature, and time. Kinetic data were fitted using pseudo-first- and pseudo-second-order and intraparticle diffusion kinetic models. The experimental findings were analyzed using the Temkin, Langmuir, Dubinin–Radushkevich, and Freundlich models. The kinetic data were well fitted by the pseudo-second-order model, and the adsorption process was well described by the Langmuir model. The functionalized activated carbon exhibited a higher maximum adsorption capacity of 175.43 mg g^−1^ for Cu(ii) removal. The Dubinin–Radushkevich model exhibited an adsorption energy of 2.620 kJ mol^−1^, indicating a physical sorption process. Overall, this study showed that activated carbon derived from *Posidonia oceanica* and functionalized with ethylenediaminetriacetic acid has the potential to act as an adsorbent for Cu^2+^ removal.

## Abstract 2

*Posidonia oceanica*, a Mediterranean marine plant, was used in this research to prepare activated carbon.

## Graphical abstract

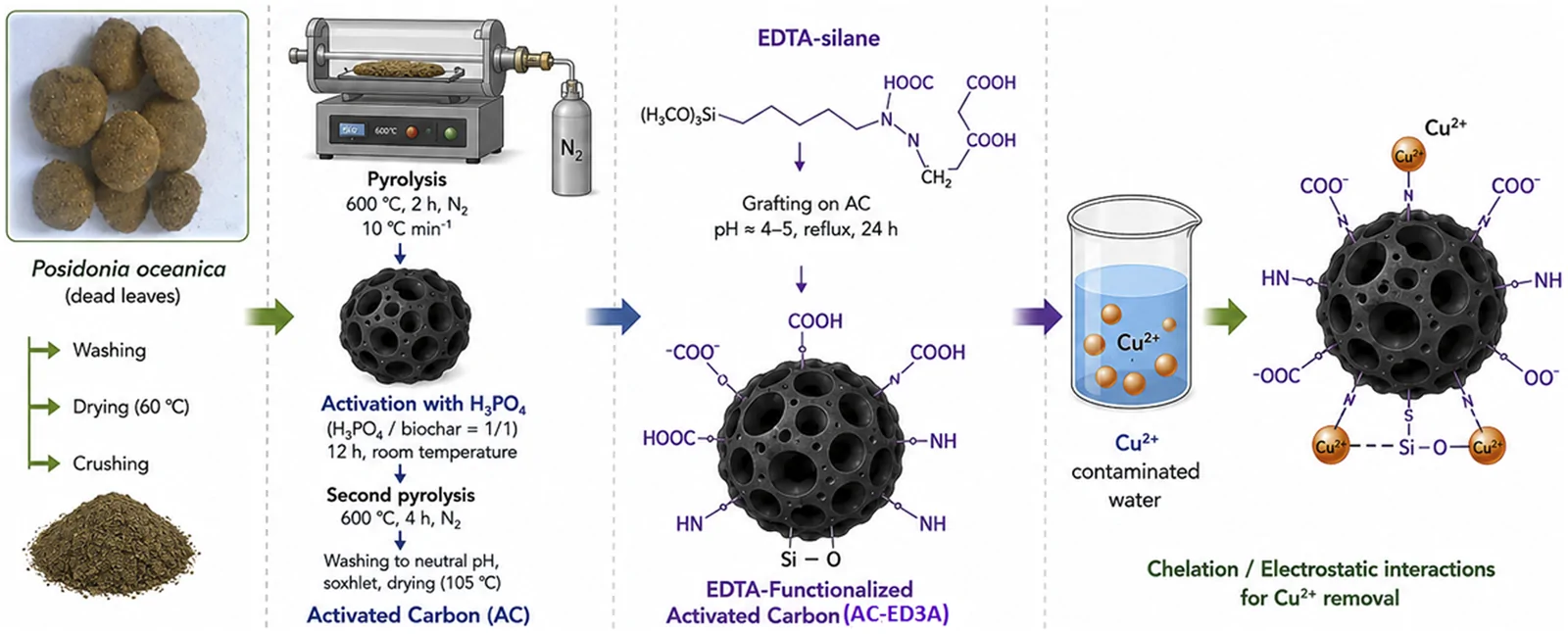

## 1 Introduction

Heavy metals are of particular concern because of their persistence and potential toxicity, even at low concentrations, and they can cause a range of adverse health effects.^1–7^ Living creatures tend to accumulate them, leading to increased concentrations across higher trophic levels.^8^ As a result, heavy metal pollutants can enter the human body through several pathways, including contaminated food and water consumption, inhalation, dermal absorption, and environmental exposure.^4,5,8–10^ The irreversibility of heavy metal contamination is the most significant characteristic that sets it apart from other types of pollution. Once released into the environment, heavy metals are difficult to remove and may persist, posing long-term risks to ecosystems and human health.^5,9–11^ Copper, which is derived from various industrial processes including mining, fertilizer manufacturing, printing, and textile productions,^3,10,12,13^ is the subject of this study. Various methods have been used for the removal of this contaminant from wastewater.^1,13–20^ Adsorption is considered an excellent alternative for removing metals from wastewater compared to other methods that are available.^14–18^ A large number of well-known adsorbent materials are available for purchase. Activated carbons are considered promising since they are inexpensive, readily available, simple to use, cost-effective, and highly reusable. Activated carbons are by far the most widely produced and industrially utilized adsorbents. Activated carbons are typically derived from non-traditional materials, particularly plant waste products such as lignite, peat, and oil residues.^16,17,21,22^ However, as these resources are becoming depleted, scientists are encouraged to investigate renewable resources that are derived from biomass.^16,17,19,22–25^ The surface modification of activated carbon through oxidation or functionalization reactions can improve its adsorption characteristics. Oxidation enables the formation of oxygenated functional groups on the carbon surface. Functionalization leads to the formation of organic groups. One of the agents used in the functionalization of different adsorbent materials is ethylenediaminetetraacetic acid (EDTA). Fe_3_O_4_/activated carbon nanocomposites functionalized with EDTA were developed for the treatment of water contaminated with metal ions, and the resulting adsorbent exhibited rapid and efficient adsorption of Cd^2+^, Pb^2+^ and As^3+^, with adsorption capacities of 87.2, 74.2 and 114.9 mg g^−1^, respectively.^26^ The authors demonstrated that the incorporation of EDTA functionalities onto activated carbon improved the interaction with metal ions and enhanced the adsorption performance.^26^ Similarly, Nguyen *et al.*^27^ used EDTA to develop an EDTA/chitosan/bagasse activated carbon composite to improve Pb^2+^ removal, achieving an adsorption capacity of 54.97 mg g^−1^. In another study, Babu *et al.*^25^ showed that the functionalization of activated carbon with EDTA also improves the adsorption capacity of activated carbon for rare earth metals in aqueous solutions. The functionalization was carried out after the oxidation of activated carbon and confirmed by various characterization techniques. The functionalized activated carbon exhibits high adsorption capacity, especially toward Nd^3+^, with the maximum adsorption capacity of 71.42 mg g^−1^, compared with 19.08 mg g^−1^ for non-functionalized activated carbon.^25^ In this regard, the goal of this study is to investigate the use of *Posidonia oceanica* to make activated carbon (AC). The activated carbon is then functionalized with *N*-[(3-trimethoxysilyl)propyl]-ethylenediaminetriacetic acid to form ethylenediaminetriacetic acid-functionalized activated carbon (AC-ED3A), which is used to adsorb Cu^2+^.

## 2 Materials and methods

### 2.1 Chemicals and *Posidonia oceanica*

Dead *Posidonia oceanica* leaves were collected from the Sousse coast in Tunisia. The collected *Posidonia oceanica* leaves were washed with water, dried at 60 °C for 24 h, and pulverized using a crusher machine.

All chemicals used were of analytical quality, were obtained from Sigma-Aldrich or Fluka, and were utilized without additional purification.

### 2.2 Preparation of activated carbon

A specific amount of powdered *Posidonia oceanica* was added to a stainless-steel reactor and subjected to heating in a horizontal furnace at a rate of 10 °C per minute under a nitrogen atmosphere for 2 hours at a temperature of 600 °C. The biochar was subsequently impregnated with a phosphoric acid solution (H_3_PO_4_/biochar = 1/1) for 12 hours at room temperature (approximately 25 °C) and then dried in an oven at 105 °C for 12 hours. The resulting biochar was subjected to a secondary pyrolysis at 600 °C for 4 hours in a nitrogen atmosphere. The activated carbon obtained was washed with distilled water until it was pH-neutral and then further washed using a Soxhlet extractor. The material was subsequently dried at 105 °C. The activated carbon was crushed, and the fraction with a particle size of 250–500 µm was recovered and stored in a desiccator until it was required for use.

### 2.3 Functionalization of activated carbon by EDTA

The synthesis of AC-ED3A was conducted following the procedure outlined by Babu *et al.*^25^ Five grams of activated carbon and sixty milliliters of nitric acid were added to a flask, and the resulting mixture was refluxed for 5 hours. The solid was filtered and washed with deionized water until achieving a neutral pH, followed by drying in an oven. Following this, 2 g of the resultant carbon was combined with 400 mL of isopropyl alcohol and water, and the pH was 4–5. The mixture was homogenized to achieve a uniform dispersion. Subsequently, 10 mL of *N*-[(3-trimethoxysilyl)propyl]-ethylenediaminetriacetic acid was added to the solution in a dropwise manner. The solution was treated with 2 mL of acetic acid and stirred for 24 hours at room temperature. The solid was filtered, rinsed with water, and dried in an oven.

### 2.4 Sorption experiments

Experiments were conducted in flasks using a constant mass of the adsorbent (0.1 g) and various starting concentrations of Cu^2+^ (30–1600 mg L^−1^). The system was shaken at 25 °C ± 2 °C; after reaching equilibrium, the supernatant was analyzed using a flame atomic absorption spectrophotometer. 0.1 mol L^−1^ NaOH or HNO_3_ solutions were used to regulate the pH value. The amount of Cu^2+^ adsorbed was quantified by eqn (1):1
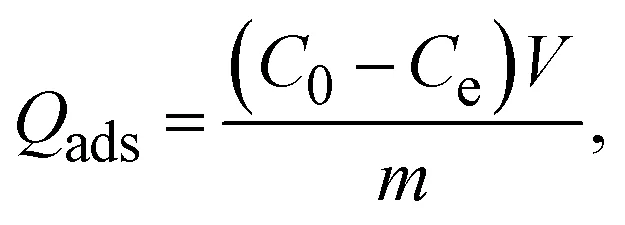
where *Q*_ads_ (mg g^−1^) is the adsorption capacity of Cu^2+^; *C*_e_ and *C*_0_ (mg L^−1^) denote the equilibrium and initial concentrations, respectively; *V* (L) is the volume of the solution; and *m* (g) is the mass of the adsorbent.

The effect of pH was studied by mixing 20 mL of a Cu^2+^ solution of 800 mg L^−1^ with 0.1 g of AC-ED3A in the pH range of 1–4. The solutions were stirred for 120 min.

The effect of the adsorbent mass on Cu^2+^ adsorption was tested by changing the adsorbent mass from 20 to 300 mg for 20 mL samples (800 mg L^−1^).

The effect of temperature was tested under the following circumstances: 120 min contact time, pH 4, 0.1 g of the adsorbent, and varying temperatures (298, 308, and 318 K). Thermodynamic parameters were expressed by the following equations:

2 Δ*G*° = −*RT* ln *K*_d_,

3
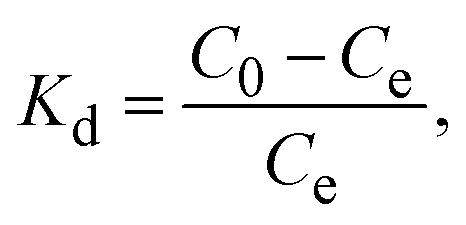
4
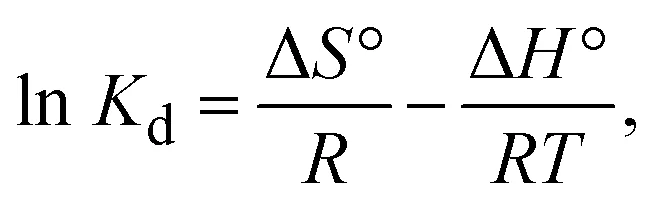
where *R* is the gas constant (8.314 J mol^−1^ K^−1^), *T* is the absolute temperature (K) and *K*_d_ is the equilibrium constant. Δ*H*° and Δ*S*° were determined by plotting ln *K*_d_*versus* 1/*T*.

All experiments were conducted in triplicate; the reported values represent the means of three independent measurements. The agreement between the mathematical models and experimental points was evaluated using the nonlinear analysis, chi-square test (*χ*^2^, eqn (5)), residual root-mean-square error (RMSE, eqn (6)), and normalized standard deviation (Δ*q*, eqn (7)).5
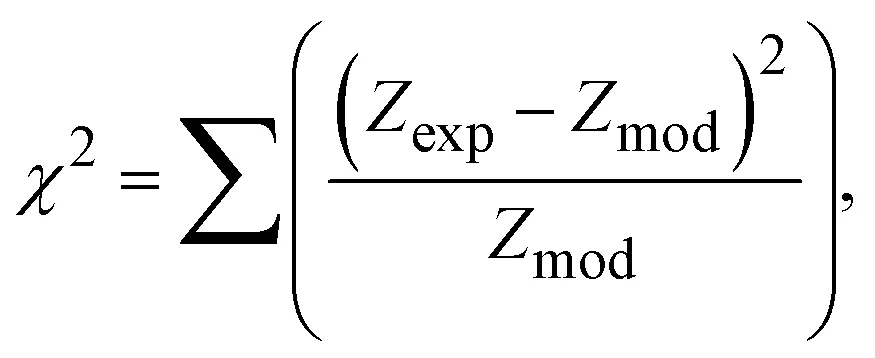
6
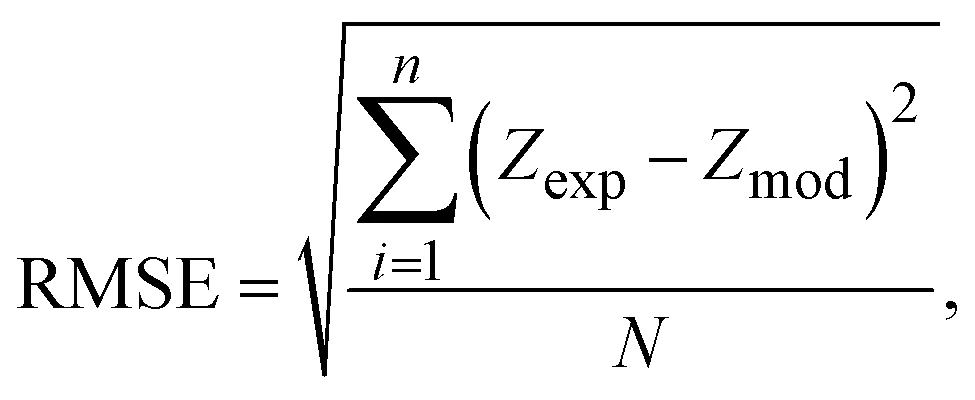
7
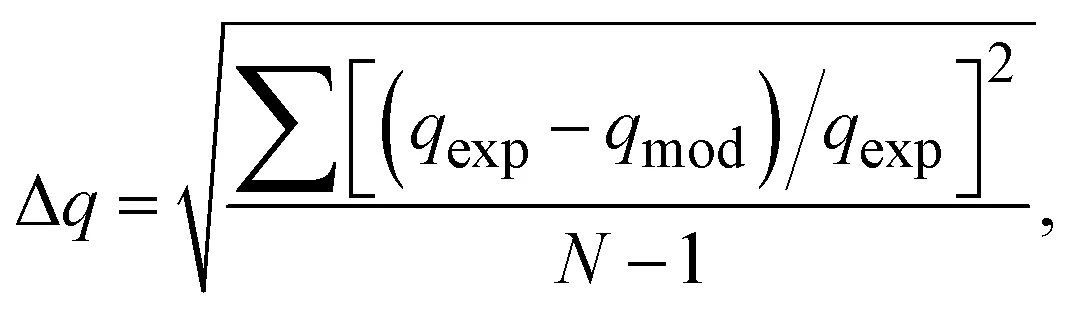
where *Z*_exp_ is the experimental value, *Z*_mod_ is the value calculated from the model, and *N* is the number of experimental data points.

### 2.5 Characterization

The point of zero charge (pH_PZC_) was estimated as reported earlier.^17,28,29^

FTIR analysis was performed using an FTIR-8400S Shimadzu spectrophotometer between 4000 and 500 cm^−1^.

Scanning electron microscopy (SEM) micrographs were acquired using a Zeiss ULTRA55 SEM microscope.

The surface area (*S*_BET_) and pore structure properties of the carbon were measured by nitrogen adsorption–desorption studies at 77 K using a Micromeritics ASAP 2020 device.

## 3 Results and discussion

### 3.1 Speciation diagram of Cu^2+^

The Hydrochemical Equilibrium-Constant Database (HYDRA) software was used to detect the ionic forms of Cu^2+^ in a solution. The speciation diagram was simulated for the Cu^2+^/NO_3_^−^/H_2_O system at 1.6 mg L^−1^ Cu^2+^ (Fig. 1).

**Fig. 1:**
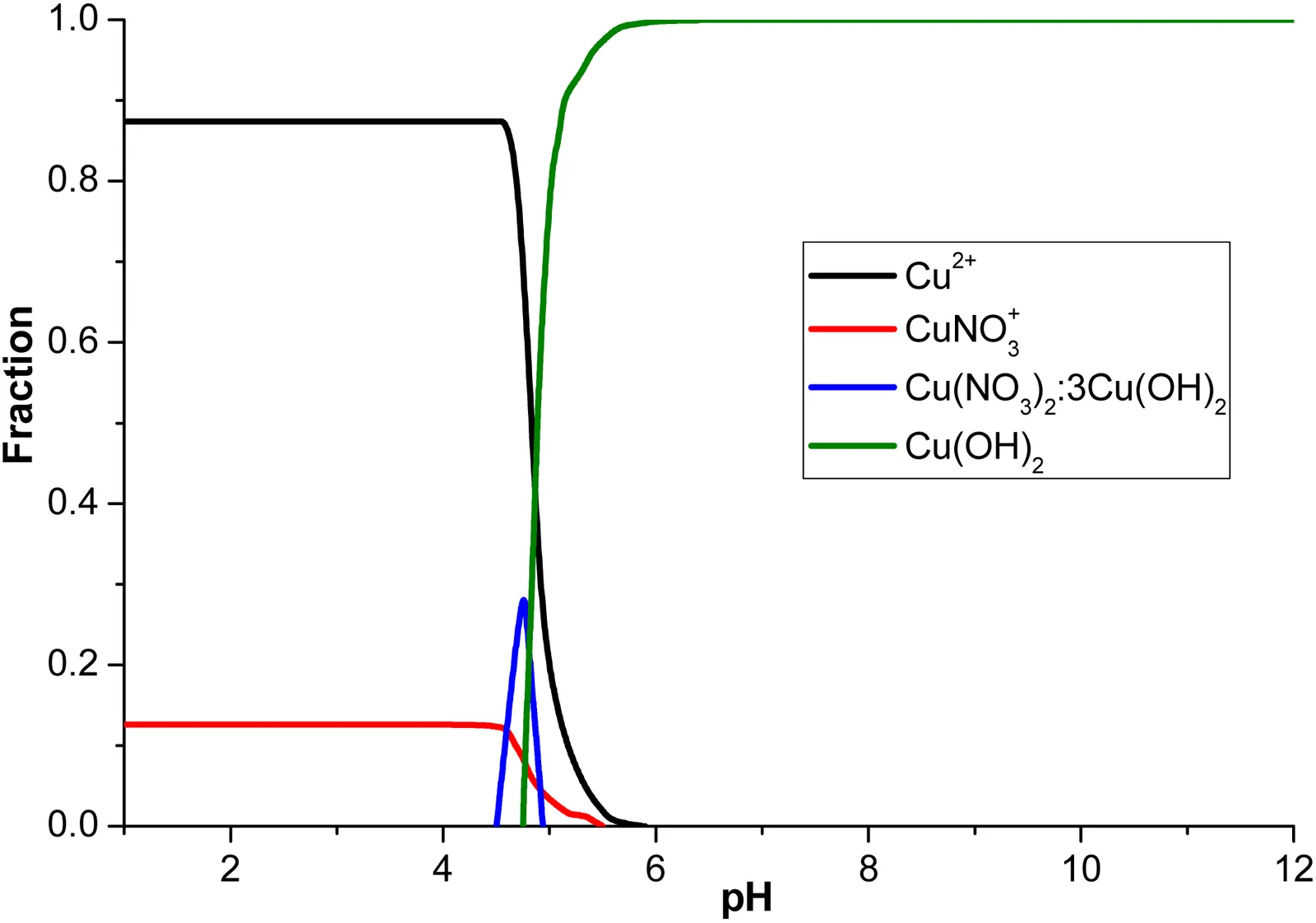
Speciation diagram of Cu^2+^ (1.6 mg L^−1^) in and NO_3_^−^ aqueous solution (50 mmol L^−1^) as a function of pH.

At acidic pH (0–4.5), the dominant form of copper is the soluble form, Cu^2+^, whereas at pH > 6, almost all of the copper is transformed into insoluble CuO. Therefore, during this work, it was decided to work at pH < 4.5.

### 3.2 Characterization of the adsorbent

FTIR measurements were used to find out which functional groups were present on the surface of AC and AC-ED3A (Fig. 2). The FTIR spectrum of AC reveals bands at 1100–1380, 1580, and 1742 cm^−1^, caused by the stretching vibrations of the C=O, C=C, and C=O groups, respectively. The broad band at 3400 cm^−1^ corresponds to –OH vibrations, which are needed for the subsequent functionalization reactions to happen. The FTIR spectrum of AC-ED3A (Fig. 2) has a peak around 1150 cm^−1^ that is caused by the stretching vibrations of the Si–O bonds.

**Fig. 2:**
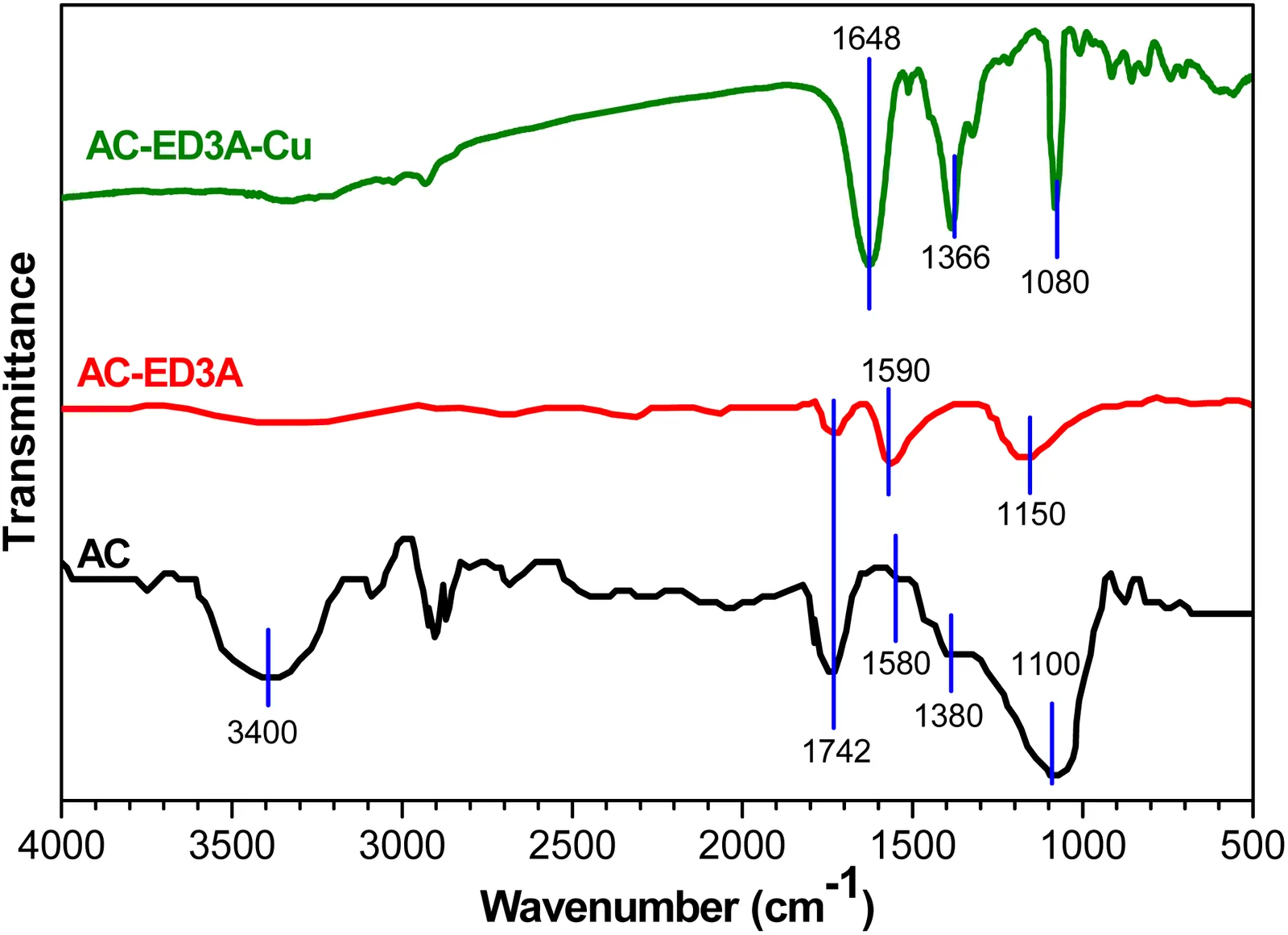
FTIR spectra of AC, AC-ED3A and AC-ED3A-Cu.

The band around 1590 cm^−1^ can be attributed to the asymmetric stretching mode of carboxylate groups of EDTA. The band around 1742 cm^−1^ corresponds to a protonated carboxylic acid.

After Cu^2+^ adsorption, the shifting of the bands from 1742 cm^−1^ to 1648 cm^−1^ and 1150 cm^−1^ to 1080 cm^−1^ and the appearance of a band at 1366 cm^−1^ reveal the involvement of the related functional groups in the adsorption process.^12,30^

As can be seen from the SEM images in Fig. 3, particles appear to be of different sizes and do not show a distinguishing shape. The morphology of AC is slightly changed following functionalization by EDTA, presenting some aggregation and a coating of lighter particles on the carbon particles. This verifies the immobilization of EDTA on the AC *via* silica bonds. The activated carbon has a specific surface area of 363 m^2^ g^−1^ (Table 1).

**Fig. 3:**
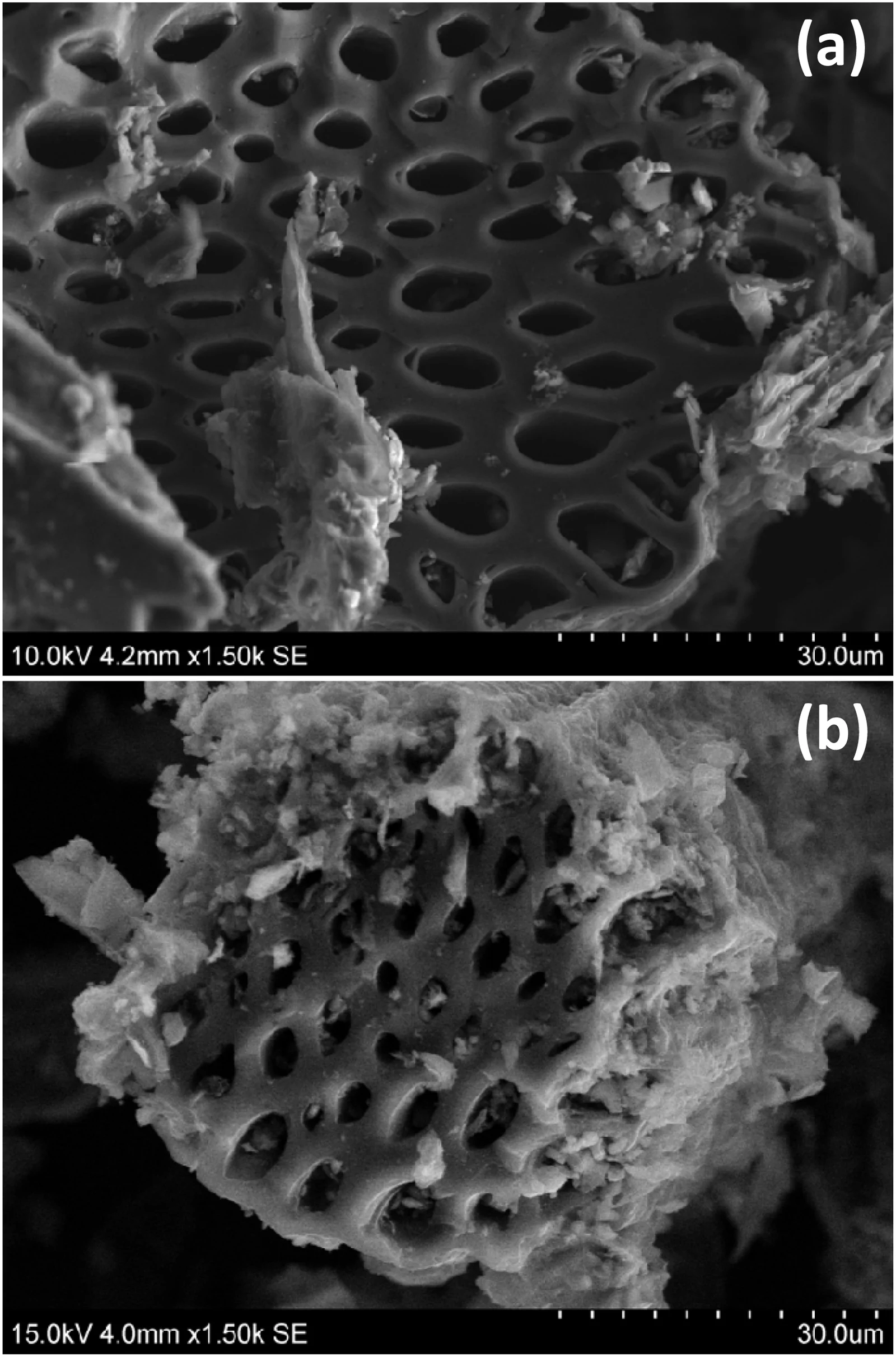
SEM images of AC (a) and AC-ED3A (b).

**Table 1:** Principal properties of activated carbon

	AC	AC-ED3A
Specific surface area, *S*_BET_ (m^2^ g^−1^)	363	346
Total pore volume (cm^3^ g^−1^)	0.026	0.024
Pore diameter (Å)	45.95	45.81
pH_PZC_	6.5	3.1

The N_2_ adsorption–desorption isotherms of the unfunctionalized and functionalized activated carbons are presented in Fig. 4.

**Fig. 4:**
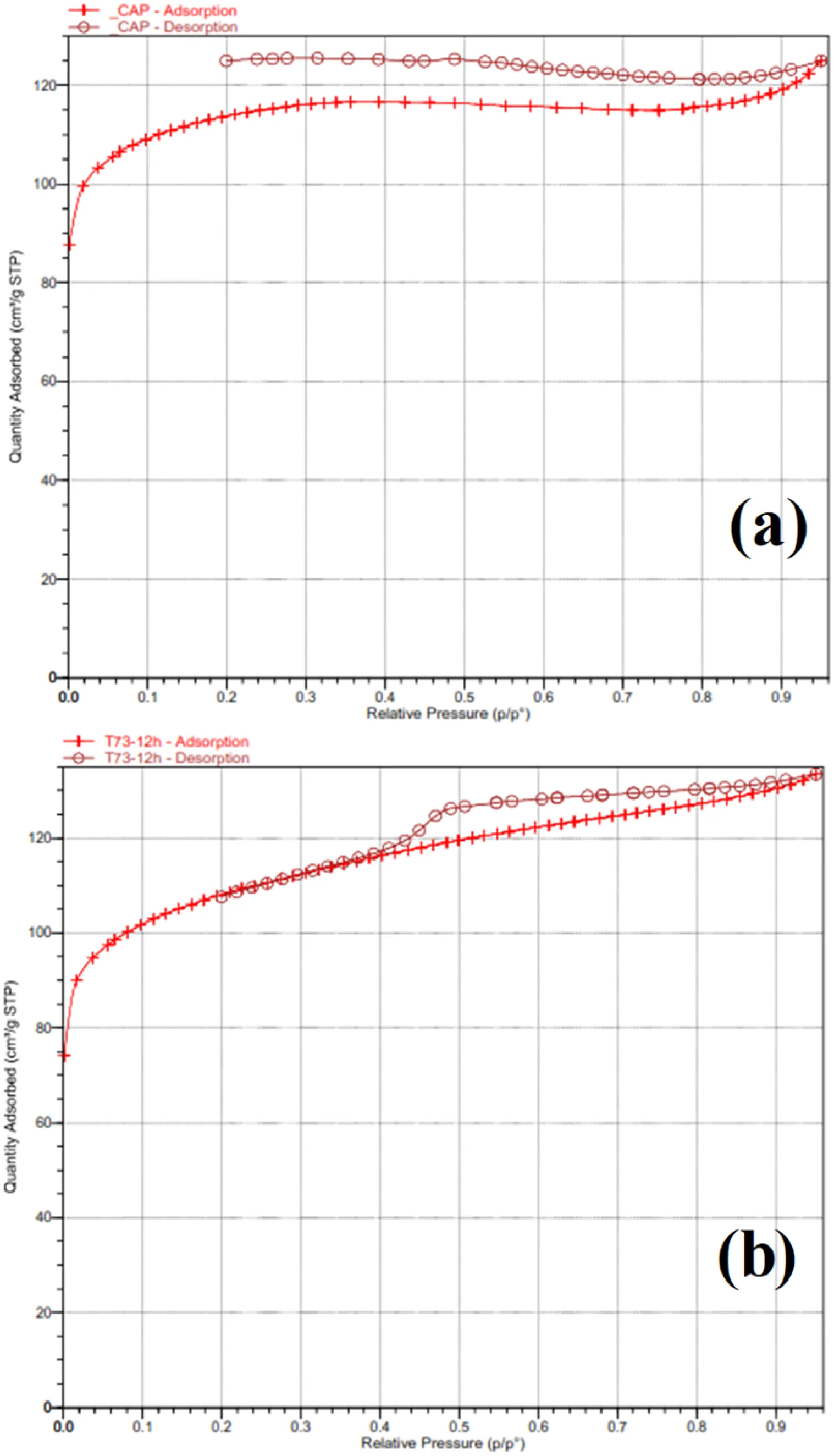
N_2_ adsorption–desorption isotherms of AC (a) and AC-ED3A (b).

Both samples show N_2_ adsorption at low relative pressures, indicating the presence of a microporous fraction, and an adsorption–desorption hysteresis at higher relative pressures, indicating a contribution of larger pores.^31,32^ Thus, the materials can be considered micro-mesoporous, as observed for activated carbons with combined type I/IV characteristics and an H4-type hysteresis.^31–33^ The textural parameters further indicate that functionalization slightly alters the pore structure. The BET specific surface area decreases from 363 to 346 m^2^ g^−1^, and the total pore volume decreases from 0.026 to 0.024 cm^3^ g^−1^. Conversely, the average pore diameter is not significantly different (45.95 and 45.81 Å for activated carbon and functionalized activated carbon, respectively). The slight decrease in the specific surface area and pore volume is attributed to the partial filling or the reduced accessibility of some pores after the surface functionalization. These pores provide sites, allowing adsorbate molecules to penetrate the cavities on the surface of the AC-ED3A.

### 3.3 Adsorption outcomes of Cu(ii) on AC-ED3A

#### 3.3.1 Cu(ii) adsorption

The sorption isotherms (Fig. 5) illustrate a strong affinity of Cu^2+^ toward the AC and AC-ED3A at low concentrations. For both materials, the adsorption capacity increases sharply at low concentrations, indicating high availability of active adsorption sites and a strong driving force for mass transfer.

**Fig. 5:**
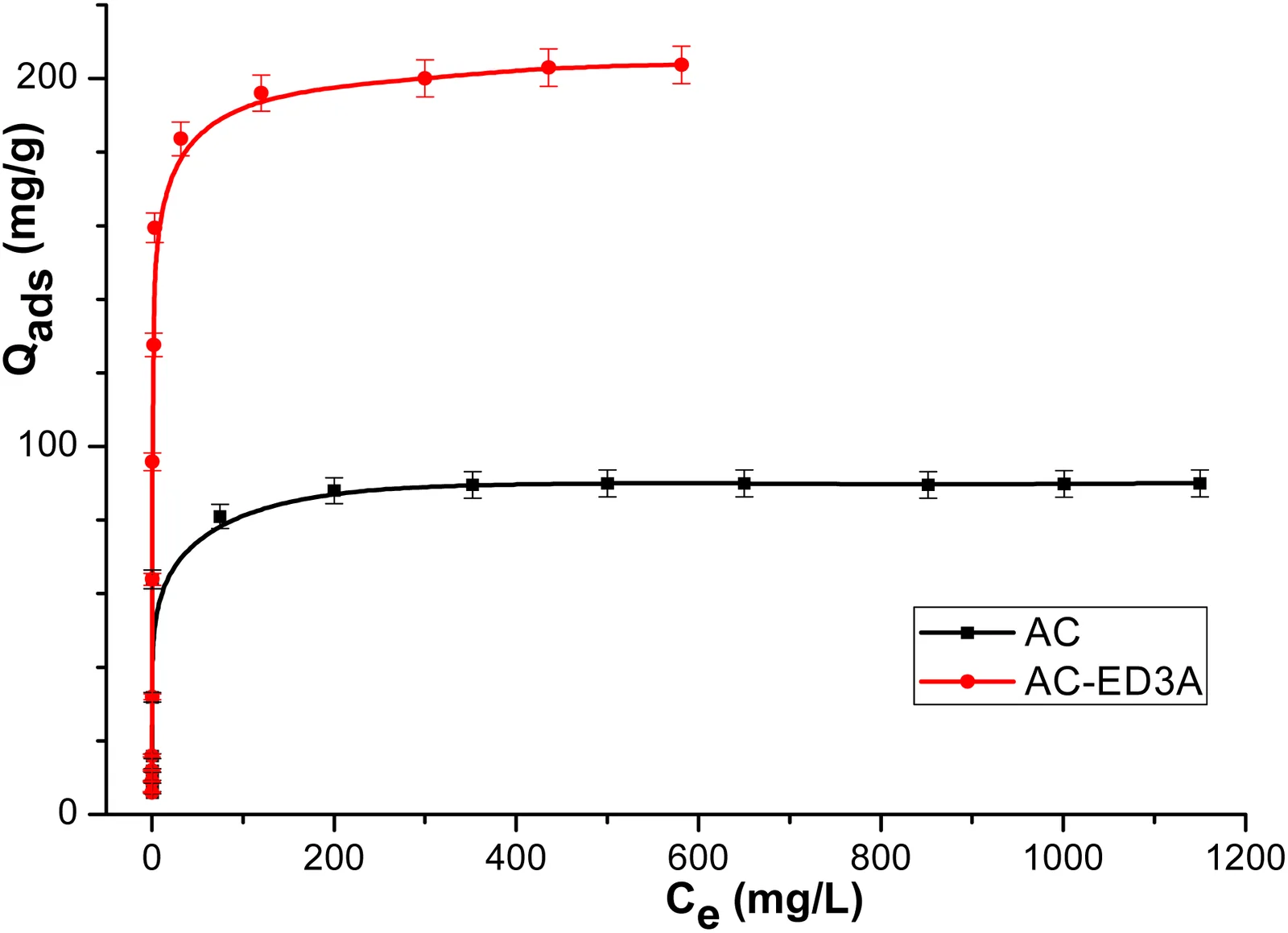
Adsorption isotherm of Cu^2+^ onto AC and AC-ED3A.

With increasing concentration of Cu^2+^, the curves gradually reach a plateau, reflecting the progressive saturation of adsorption sites and the establishment of equilibrium. AC-ED3A exhibits a significantly higher adsorption capacity than AC (Fig. 5). This can be attributed to the introduction of EDTA functional groups, which provide additional chelating sites and increase the affinity toward Cu^2+^ ions through coordination/chelation or/and electrostatic interactions. The rapid attainment of a higher plateau for AC-ED3A suggests a more homogeneous distribution of adsorbent sites and stronger adsorbate–adsorbent interactions. It is important to note that the small changes in textural properties (Table 1 and Fig. 4) are worth mentioning because they indicate that the increase in the Cu^2+^ adsorption capacity of the functionalized carbon is not related to an increase in the specific surface area or pore volume, but is related to the chemical modification of the carbon surface. Therefore, AC-ED3A is chosen as the adsorbent for the subsequent studies on the effects of the pH and temperature and adsorption isotherms.

#### 3.3.2 Effect of pH

The impact of pH was studied in the pH range from 1 to 4 using AC-ED3A as the adsorbent (Fig. 6).

**Fig. 6:**
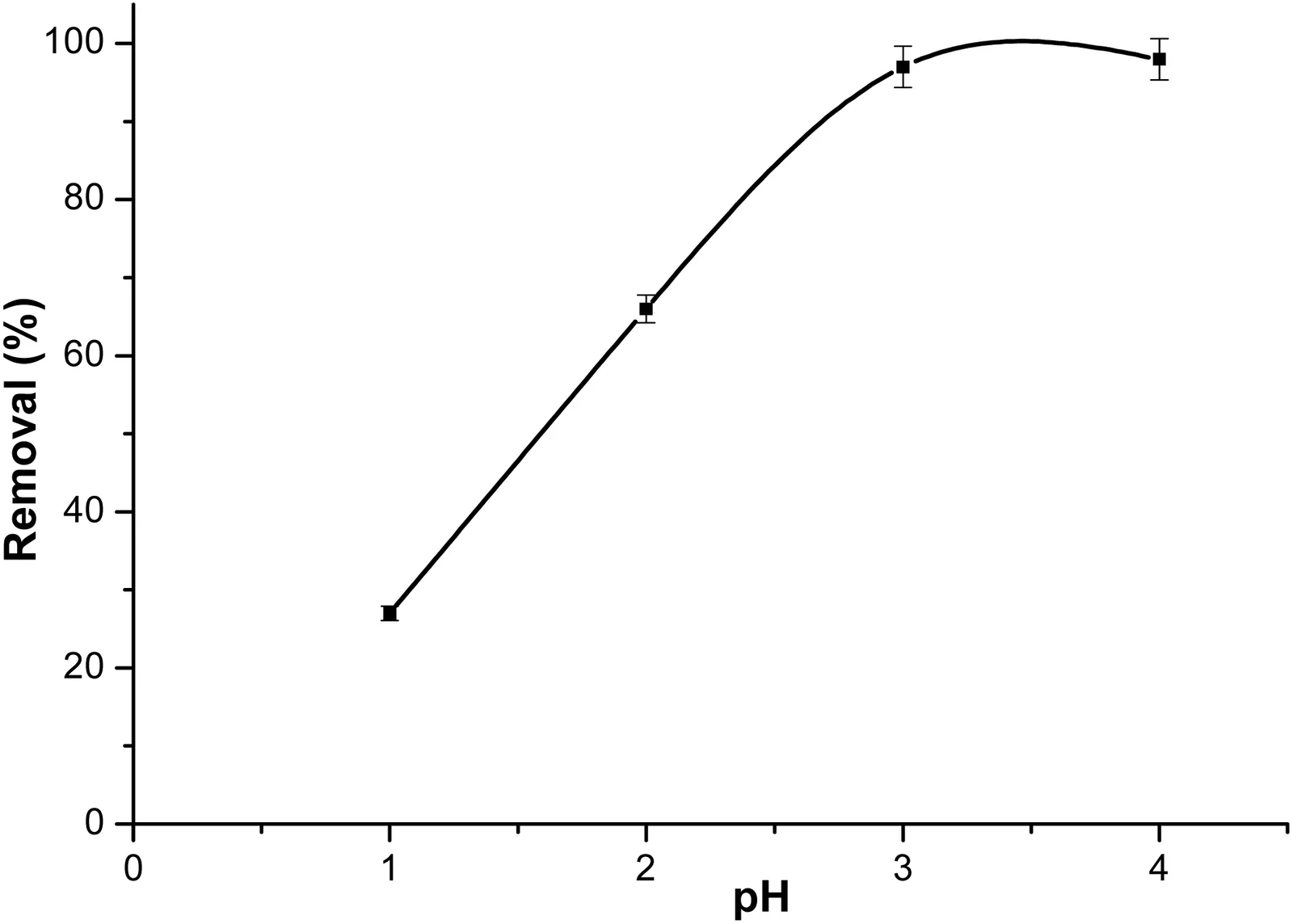
Effect of pH on the adsorption of Cu(ii) onto AC-ED3A.

According to the speciation diagram, at a pH higher than 4.5, the formation of the complex Cu(NO_3_)_2_:3Cu(OH)_2_ and the crystal CuO occurs. The removal efficiency of Cu^2+^ is low in strongly acidic media, only about 27% at pH 1, and then increases to 65% at pH 2 (Fig. 6). This is explained by the competition between Cu^2+^ and H^+^ ions in strongly acidic media. At pH 3, the removal rate is about 97% and reaches 98% at pH 4. It appears that a pH between 3 and 4 corresponds to the maximum adsorption of Cu^2+^ on AC-ED3A. Therefore, in subsequent studies, the experiments were carried out at a solution pH of 3–4.

#### 3.3.3 Effect of the mass of the adsorbent

An increase in Cu^2+^ removal is observed as the mass of the adsorbent increases from 20 to 100 mg (Fig. 7). This improvement can be attributed to the increase in the number of adsorption sites. For adsorbent doses higher than 100 mg/20 mL, the removal of Cu^2+^ remains constant. Adsorption isotherms were studied with an adsorbent mass of 100 mg.

**Fig. 7:**
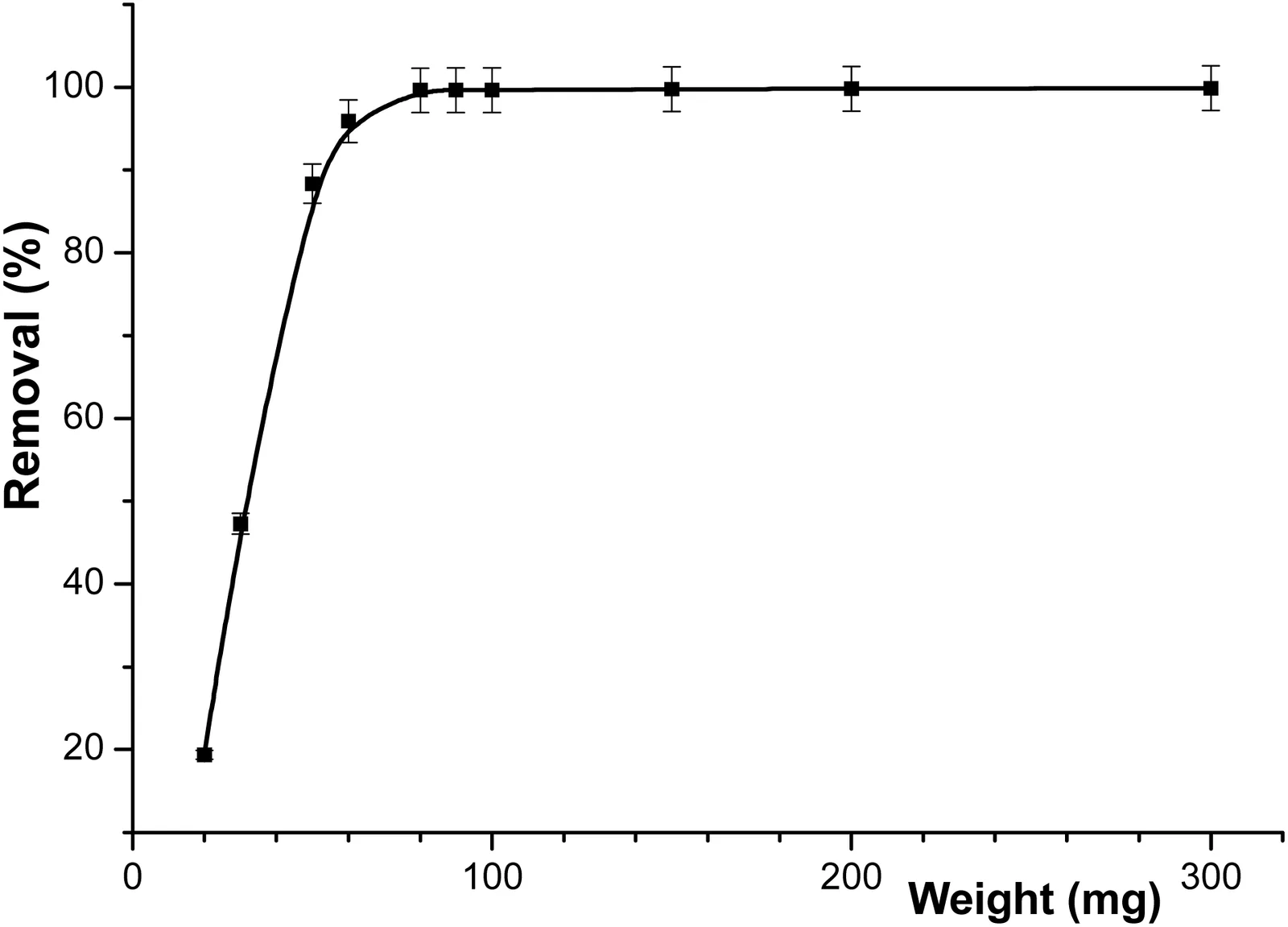
Effect of the mass of AC-ED3A on the adsorption of Cu^2+^.

#### 3.3.4 Effect of temperature

Thermodynamic parameters were employed to figure out the feasibility of the adsorption process (Table 2 and Fig. 8). The free energy values are negative, indicating the spontaneous adsorption behavior of Cu^2+^ ions on AC-ED3A. The negative Δ*G*° values decrease with an increase in temperature, which shows a decrease in the amount adsorbed. The negative enthalpy (Δ*H*° = −149.78 kJ mol^−1^) implies an exothermic process. Also, the entropy (Δ*S*° = −456 J mol^−1^) of <0 shows that the disorder has decreased.^12,28^

**Table 2:** Thermodynamics of the adsorption of Cu^2+^ onto AC-ED3A

Temperature (K)	Δ*G*° (kJ mol^−1^)	Δ*H*° (kJ mol^−1^)	Δ*S*° (J mol^−1^ K^−1^)
298	−13.81	−149.78	−456
308	−9.24		
318	−4.68		

**Fig. 8:**
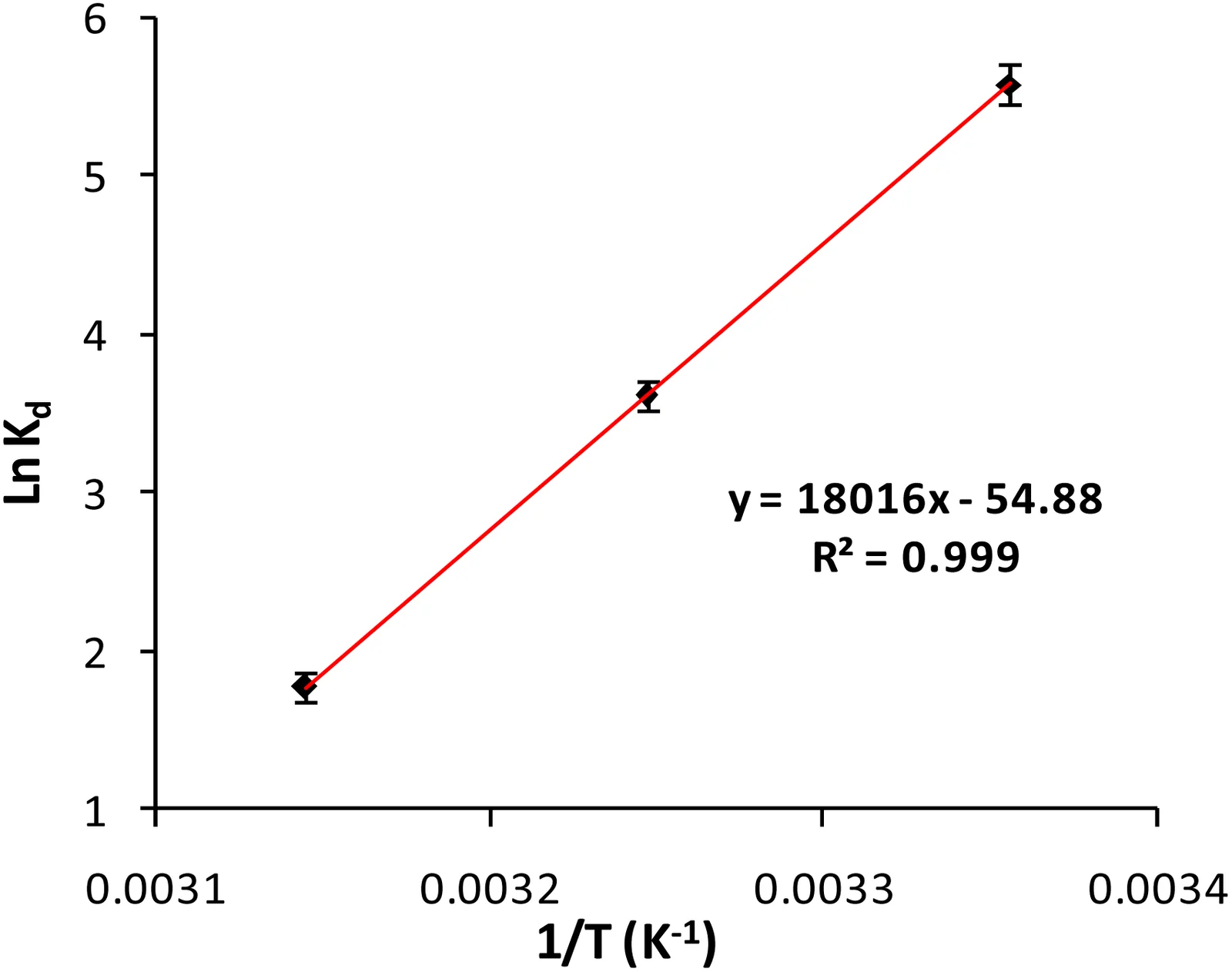
Plot of ln(*K*_d_) *versus* 1/*T*.

#### 3.3.5 Adsorption kinetics

Fig. 9 demonstrates the impact of contact time on the percentage of Cu^2+^ adsorption. The adsorption percentage increases rapidly in the first few minutes and reaches nearly 99.6% after 45 min.

**Fig. 9:**
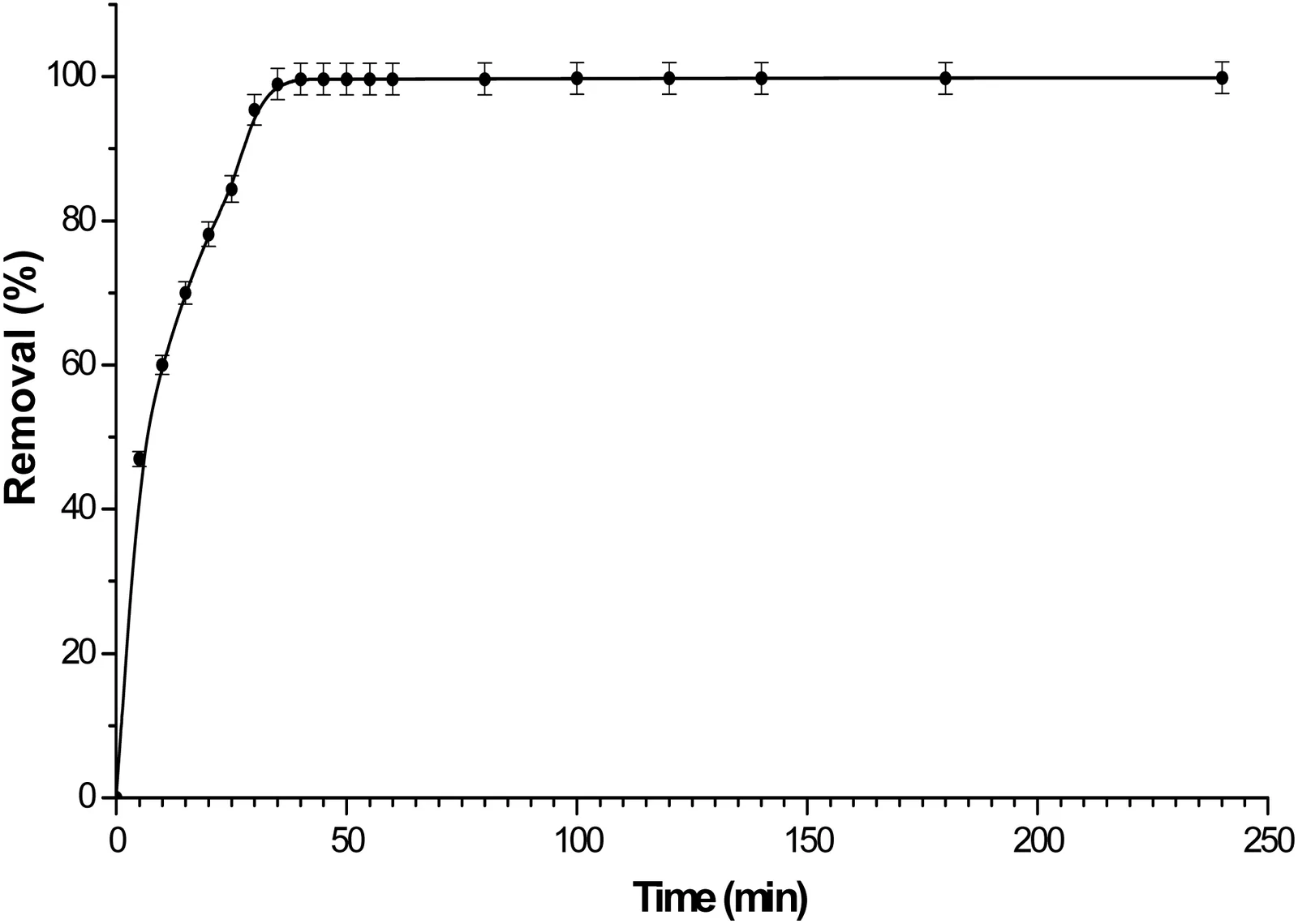
Effect of the contact time on the adsorption of Cu^2+^.

This evolution agrees with the progressive occupation of available active sites on the AC-ED3A surface, which is initially favored by the presence of plenty of free sites and a steep concentration gradient. The percentage of adsorption is almost constant after 45 minutes, which means that adsorption equilibrium is attained, and this shows that the active sites on AC-ED3A are completely saturated. Therefore, a 45 minutes contact time can be considered as the optimum time for the maximum removal of Cu^2+^.

The pseudo-first-order (eqn (8)) and pseudo-second-order (eqn (9)) kinetic models were employed to comprehend the mechanism of the adsorption process.

8 ln(*Q*_ads_ − *Q*_*t*_) = ln *Q*_ads_ − *K*_1_*t*,

9
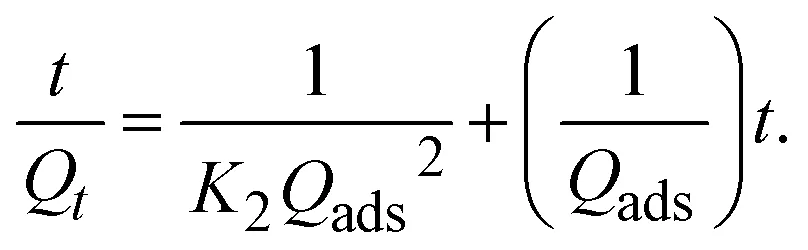


Kinetic analysis (Table 3) shows that the P-SO model describes the data from experiments better than the P-FO model. Indeed, the P-SO model's correlation coefficient (*R*^2^ = 0.99) is much greater than that of the P-FO model (*R*^2^ = 0.82). Furthermore, the adsorption capacity calculated by the P-SO model (166.56 mg g^−1^) is very close to the experimental value (159.67 mg g^−1^), whereas that obtained with the P-FO model (124.05 mg g^−1^) deviates considerably (Table 3). The lower values of *χ*^2^, Δ*Q*, and RMSE for the P-SO model also confirm its better fit. These results indicate that the P-SO kinetic model is better for describing the adsorption of Cu^2+^ onto AC-ED3A.

**Table 3:** Kinetics of the adsorption of Cu^2+^ on AC-ED3A

Pseudo-first-order (P-FO)		Pseudo-second-order (P-SO)	
*Q*_exp_ (mg g^−1^) 159.67			
*Q*_cal_ (mg g^−1^)	124.05	*Q*_cal_ (mg g^−1^)	166.56
*k*_1_ (min^−1^)	0.1049	*k*_2_ (g mmol^−1^ min^−1^)	0.0013
*R* ^2^	0.82	*R* ^2^	0.99
*χ* ^2^	134.76	*χ* ^2^	118.92
Δ*Q*	0.0752	Δ*Q*	0.0651
RMSE	42.97	RMSE	35.19

To better understand the reaction sequence and anticipate the rate-limiting step of the adsorption process, the intraparticle diffusion model, given by eqn (10), was used.

10 *Q*_*t*_ = *K*_id_*t*^0.5^ + *C*_st_

The graphical representation of *Q*_*t*_*versus t*^0.5^ (Fig. 10) displays two linearities, illustrating that Cu(ii) adsorption onto AC-ED3A is governed by at least two mechanisms. In fact, the high *R*^2^ values of the linear relationship of the plots suggest that intraparticle diffusion plays a role in the adsorption.

**Fig. 10:**
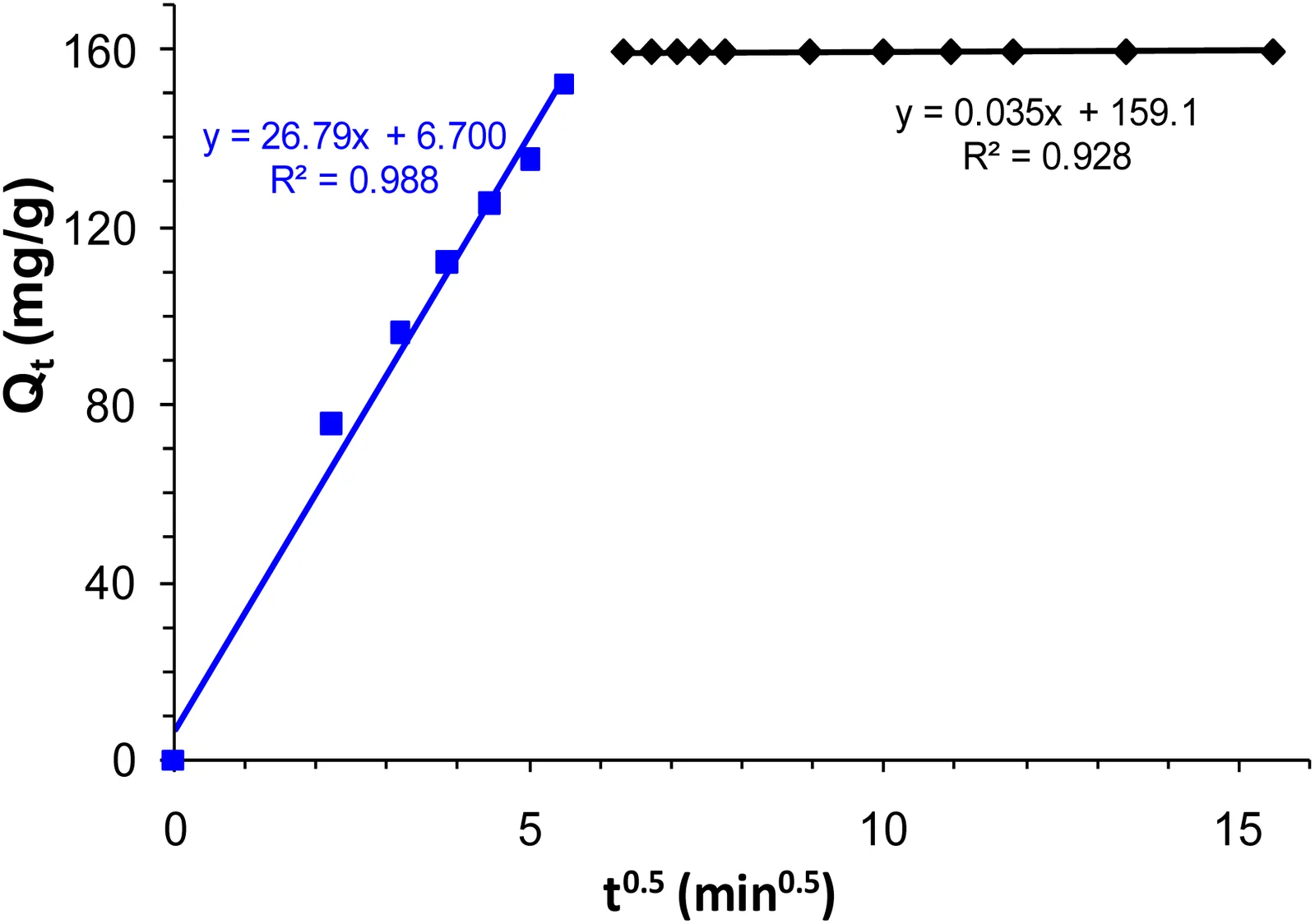
Intraparticle diffusion plot of Cu^2+^ adsorption on AC-ED3A.

#### 3.3.6 Adsorption isotherms

The adsorption capacity of AC-ED3A was evaluated using Dubinin–Radushkevich, Freundlich, Langmuir, and Temkin isotherms. Their linear forms are exhibited, respectively, in eqn (11)–(14). The parameters pertinent to the adsorption models are enumerated in Table 4.

11 ln(*Q*_ads_) = ln(*Q*_e_) − *βε*^2^,

12
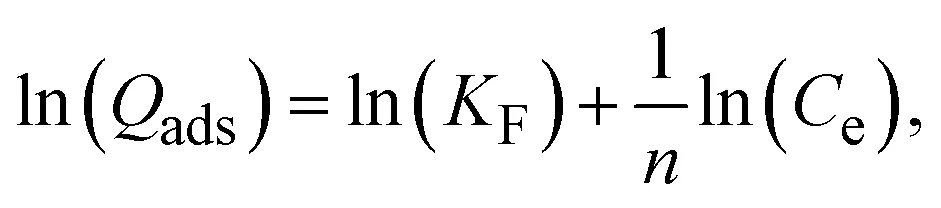
13
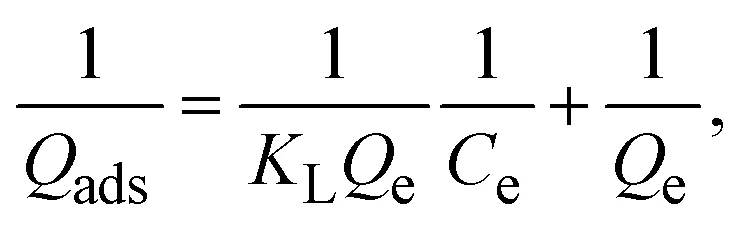

14 *Q*_ads_ = *B* ln(*A*_T_*C*_e_).

**Table 4:** Isotherm constants for the adsorption of Cu^2+^ on AC-ED3A

Langmuir	*Q*_e_ (mg g^−1^)	175.43
	*K*_L_ (L mg^−1^)	0.73
	*R*_L_	0.0008–0.0436
	*R* ^2^	0.97
	*χ* ^2^	53.73
	Δ*Q*	0.0835
	RMSE	20.87
Temkin	*A*_T_ (L g^−1^)	22.12
	*B*	24.61
	*R* ^2^	0.91
	*χ* ^2^	90.21
	Δ*Q*	0.2011
	RMSE	23.93
Dubinin–Radushkevich	*β* (mol^2^ kJ^−2^)	0.0728
	*Q*_e_ (mg g^−1^)	147.62
	*R* ^2^	0.87
	*χ* ^2^	134.12
	Δ*Q*	0.2717
	RMSE	33.85
Freundlich	1/*n*	0.3054
	*K*_F_	46.42
	*R* ^2^	0.74
	*χ* ^2^	437.86
	Δ*Q*	0.9475
	RMSE	58.50

The Langmuir model best correlates with the experimental data, as evidenced by the high value of the coefficient of determination (*R*^2^ = 0.97) and the smallest values of Δ*Q* (0.0835) and RMSE (20.87). This observation suggests that Cu^2+^ adsorption on AC-ED3A occurs primarily as a monolayer on a reasonably uniform surface, with several energetically comparable active sites.^34–37^

The adsorption capacity obtained from this model (*Q*_e_ = 175.43 mg g^−1^) highlights the excellent ability of AC-ED3A to adsorb Cu^2+^ ions. Furthermore, the separation factor (*R*_L_) values below 1 and close to zero confirm the highly favorable nature of the adsorption within the studied concentration range.^36–38^

The Temkin model also provides a satisfactory fit (*R*^2^ = 0.91), although it is less effective than the Langmuir model. The constants *A*_T_ (22.12 L g^−1^) and *B* (24.61 J mol^−1^) indicate a substantial affinity between Cu^2+^ and the coal surface and that the intensity of adsorption diminishes progressively with the occupancy of active sites, consistent with the assumptions of this model.^34–37^ This trend suggests the existence of [Cu^2+^]–[AC-ED3A] interactions and a nonuniform distribution of the adsorption energy on the AC-ED3A surface. However, the higher values of *χ*^2^, Δ*Q*, and RMSE show that this model describes the experimental data less accurately than the Langmuir model.

The Dubinin–Radushkevich model provides a moderate fit, with a coefficient of determination of 0.87. The adsorption capacity estimated by this model (147.62 mg g^−1^) is lower than that obtained with the Langmuir model, which shows that the real adsorption capacity of AC-ED3A is underestimated. The mean free energy of adsorption (*E* = (2*β*)^−1/2^) is found to be 2.6207 kJ mol^−1^ < 8 kJ mol^−1^, indicating a physisorption phenomenon.^36,37^ This result indicates that the Cu(ii) adsorption on AC-ED3A is mainly controlled by physical interactions such as electrostatic and van der Waals interactions.

On the contrary, the Freundlich model shows the lowest coefficient of determination (*R*^2^ = 0.74) and the highest values of *χ*^2^ (437.86), Δ*Q* (0.9475), and RMSE (58.50), which points to an inadequate fit to the experimental data. The 1/*n* value of 0.3054 falls in the typical range for favorable adsorption and suggests the good affinity of AC-ED3A for Cu^2+^ ions.^34,35,37^ The *K*_F_ constant of 46.42 also confirms a good capacity of AC-ED3A to adsorb Cu^2+^ ions. However, the poor statistical performance of this model suggests that the assumption of a highly heterogeneous surface is not suitable for AC-ED3A.

It turns out that the Langmuir model most accurately characterizes the adsorption behavior of Cu^2+^ on AC-ED3A. Furthermore, the adsorption mechanism is dominated by physical interactions, demonstrating the possibility of adsorbent regeneration for reuse in subsequent adsorption cycles.

### 3.4 Comparison of Cu^2+^ adsorption onto various sorbents

The adsorption capacity of the synthesized AC-ED3A was assessed by comparing its uptake with that of other adsorbents (Table 5).

**Table 5:** Cu^2+^ adsorption capacities onto multiple sorbents

Sorbent	pH	*T* (°C)	*C*_0_ (mg L^−1^)	Dose (g L^−1^)	*Q*_ads_ (mg g^−1^)
Berry leaf^39^	6	25	1–13	4	2.8
Banana peel powder^40^	5	30	2–10	5	5.7
Natural Jordanian zeolite^41^	6	—	100–600	20	14.3
Natural moss^42^	5	—	5–1000	5	22.7
Citric acid cross-linked chitosan and carboxymethyl cellulose hydrogel^12^	6	25	10–100	2	28.08
Fly ash-coated chitosan^43^	5	25	63–1625	10	28.6
Banana peels^44^	5.5	25	10–200	1	29.26
Bottom ash^45^	5	25	100–1000	20	32.4
Coconut shell-Fe_3_O_4_-alginate^46^	5	25	40–300	2	40.42
Activated carbon from grape bagasse^47^	5	45	10–100	2	43.47
Carbon foam from banana peel^48^	4	30	0–1000	25	49.5
Chitosan-silver nanoparticles^13^	7	25	5–100	0.2	110.67
Zwitterion-chitosan^49^	6	40	10–150	1	123.5
Fly ash^45^	5	25	100–1000	2	151.4
CA-EDTA (this study)	3–4	25	30–1600	5	175.43
Cross-linked chitosan grafted with methyl methacrylate^50^	4	30	100–600	2.5	192.31
MIL-121 onto alginate beads^51^	5	25	10–400	1	204.5
Paper-mill sludge biochar^52^	4	25	30–80	1	451.51

The obtained adsorption capacities ranged from 2.8 to 451.51 mg g^−1^ (Table 5) and were controlled by the nature and functionalization of the materials and the experimental conditions. Natural adsorbents without modification, including berry leaves, banana peel powder, natural Jordanian zeolite and natural moss, showed relatively low capacities in the range from 2.8 to 22.7 mg g^−1^, which may be attributed to the limited number of active sites and moderate affinity for Cu^2+^ ions. Chemical modifications like chitosan-silver nanoparticles (110.67 mg g^−1^), zwitterion-chitosan (123.5 mg g^−1^), and methyl methacrylate-grafted crosslinked chitosan (192.31 mg g^−1^) usually improved the adsorption performance. This improvement is linked to the greater availability of functional sites and to the introduction of groups capable of interacting more strongly with Cu^2+^. In this context, the AC-ED3A developed in the present work exhibited an adsorption capacity of 175.43 mg g^−1^ at pH 3–4 and 25 °C, with an initial concentration ranging from 30 to 1600 mg L^−1^ and an adsorbent dose of 5 g L^−1^. The pH has a strong effect on this performance because at acidic pH, the protonation of surface functional groups can competition between H^+^ and Cu^2+^ for adsorption sites; however, the high capacity obtained for CA-EDTA at pH 3–4 indicates the strong affinity of the surface functional groups of the adsorbent for Cu^2+^ ions.

The high initial concentration used in this study also increases the driving force of mass transfer and the occupancy of active sites, which may contribute to the high *Q*_ads_ value. However, the relatively high dose of AC-ED3A (5 g L^−1^) is higher than that of other materials with only 1–2 g L^−1^. The dose strongly affects the utilization of active sites and the overall performance of the process. At an ambient temperature (25 °C), AC-ED3A's capacity is still higher than that of many materials reported under similar conditions, for example, fly ash (151.4 mg g^−1^), zwitterion-chitosan (123.5 mg g^−1^), and chitosan-silver nanoparticles (110.67 mg g^−1^). It is slightly lower than cross-linked chitosan grafted on methyl methacrylate (192.31 mg g^−1^) and MIL-121 incorporated in alginate beads (204.5 mg g^−1^), while the highest value is obtained with paper-mill sludge biochar (451.51 mg g^−1^). However, note that different adsorbents were tested at different pH values, initial concentrations, doses and temperatures. Therefore, the obtained value of 175.43 mg g^−1^ for AC-ED3A at 25 °C and pH 3–4 shows a competitive adsorption capacity, particularly interesting for the removal of Cu^2+^ in acidic media, and confirms the beneficial role of the EDTA functionalization in improving the material's affinity to Cu^2+^ ions.

## 4 Conclusions

In this work, AC-ED3A was successfully synthesized and quantified for its efficacy in removing aqueous Cu^2+^ ions. The characterization confirmed the successful immobilization of EDTA on the activated carbon surface, with the preservation of its porosity and a small change in the specific surface area. The experiments showed that the adsorption performance of the functionalized activated carbon was significantly better than that of the unfunctionalized one. The optimum conditions for adsorption were a pH of 3–4, an adsorbent amount of 100 mg and a contact time of 45 min. Under these conditions, nearly complete Cu^2+^ adsorption was achieved, indicating the high affinity of AC-ED3A for copper ions. The adsorption was spontaneous and exothermic; it followed P-SO kinetics and was governed by the active sites of the adsorbent and Cu^2+^ ions' interactions. Intraparticle diffusion played a contributing role in the adsorption mechanism; however, it failed to be the sole rate-limiting step. The Langmuir isotherm best matched the experimental results, offering an adsorption capacity of 175.43 mg g^−1^, suggesting monolayer adsorption on a relatively homogeneous surface. According to the Dubinin–Radushkevich model, physical interactions dominated the adsorption process. AC-ED3A showed competitive adsorption performance for Cu^2+^ removal compared to the adsorbents reported in the literature. The presence of carboxylate and amine groups from EDTA provided more active sites and improved the binding of metal ions to a large extent. These results show that AC-ED3A is an efficient, promising and possibly regenerable adsorbent for the removal of copper from copper-contaminated wastewater and other effluents contaminated with heavy metals. Future studies should focus on regeneration and reuse cycles and adsorption in multimetal systems and in real wastewater matrices.

## Author contributions

Hanedi Elhleli: writing – original draft, methodology, investigation, formal analysis, data curation. Najib Mnasri: writing – original draft, methodology, investigation, data curation. Salah Neghmouche Nacer: validation, formal analysis, data curation. Mohammed Larbi Benamor: formal analysis, data curation, conceptualization. Ramzi Khiari: visualization, validation, methodology, supervision. Younes Moussaoui: writing – review & editing, writing – original draft, visualization, validation, methodology, supervision.

## Conflicts of interest

The authors declare that they have no known competing financial interests or personal relationships that could have appeared to influence the work reported in this paper.

## Supplementary Material

RA-OLF-D6RA06504J-s001

## Data Availability

The data supporting the findings of this study are available within the article. Supplementary information (SI): the modelization of adsorption isotherms of Cu^2+^ onto AC-ED3A. See DOI: https://doi.org/10.1039/d6ra06504j.
