# Supplementary material for: Ethylenediaminetriacetic acid-functionalized activated carbon derived from Posidonia oceanica for the removal of Cu(ii)
Source: RSC Adv. 2026 Oct 5;16(46):59968–77. doi: 10.1039/d6ra06504j (PMC13636002; doi:10.1039/d6ra06504j)

Supplementary Information (SI) for RSC Advances.
This journal is © The Royal Society of Chemistry 2026

Supplementary Information

Ethylenediaminetriacetic Acid-Functionalized Activated Carbon derived from *Posidonia oceanica* for removal of Cu(II)

Hanedi Elhleli, Najib Mnasri, Salah Neghmouche Nacer, Mohammed Larbi Benamor, Ramzi Khiari, Younes Moussaoui

Modelization of Adsorption isotherms : (a) Dubinin-Radushkevich, (b) Freundlich, (c) Langmuir, (d) Temkin

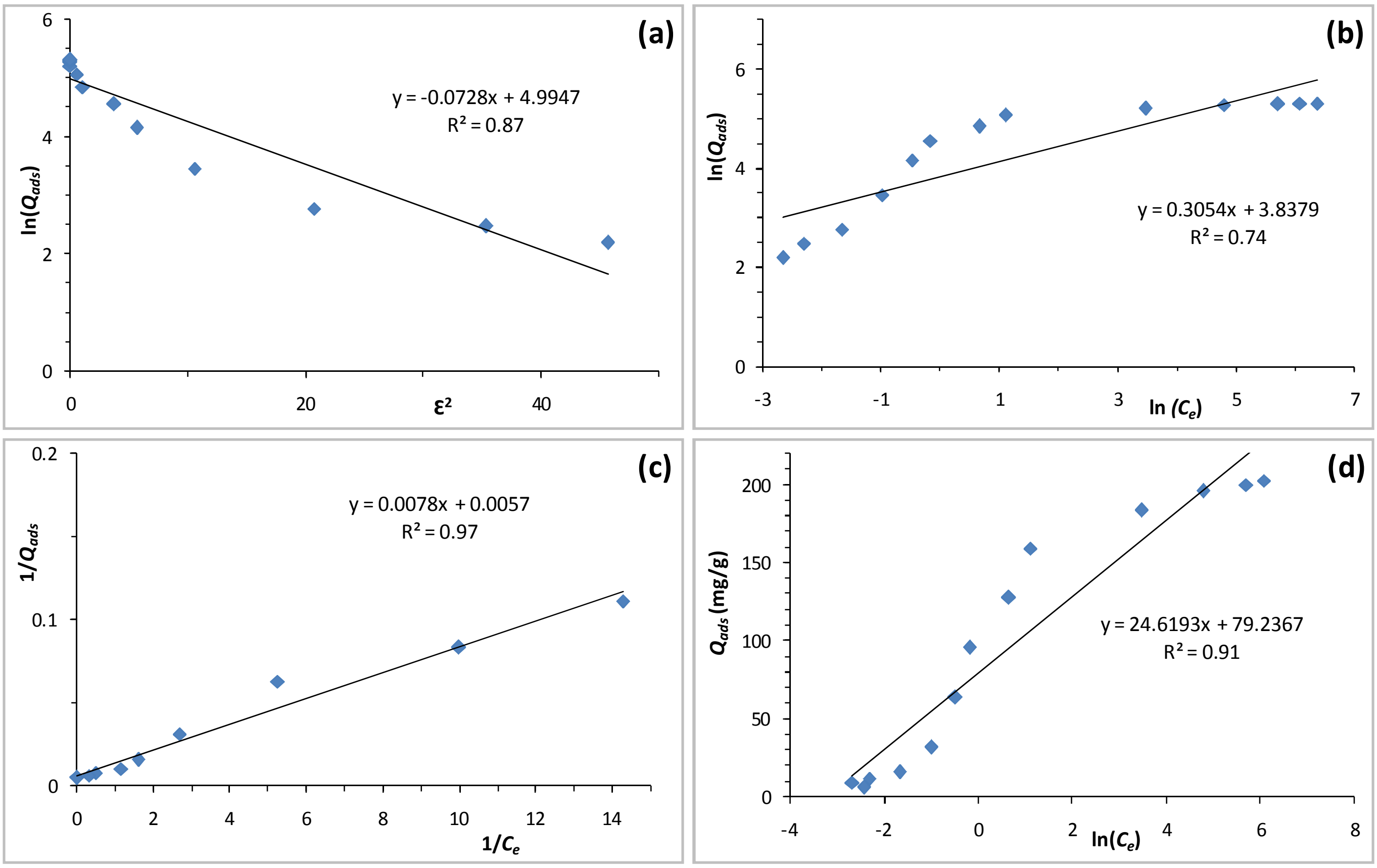

Supplement: Supplementary file 1 [file RA-016-D6RA06504J-s001.pdf]
